# Progress in Medicine

**Published:** 1902-03-15

**Authors:** 


### ROGRESS IN MEDICINE.

(*Continued from page* 389.)

The treatment of acute rheumatism in Dr. Caton's hands[8] is largely directed to prevent any endocardial mischief. He prescribes absolute rest in the recumbent position; the use of small blisters from the clavicle to the nipple, followed by small poultices as a stimulant to the centres of the first four dorsal nerves; and the internal administration of sod. iodide with the view of absorbing the material effused on the valves, if the case be seen early enough. To these indications Professor Clifford Allbutt would add the limiting of the fluids in the food.

The bacteriological causation of acute rheumatism has been variously attributed to:—

1. A diplococcus, by Von Leyden, Triboulet, Dana,

Apert, Wasserman and Malakoff, FitzMeyer and Poynton, and Paine.[9]

2. A specific anærobic bacillus, by Achalme, Thiroloix and others.

3. Various infections, without any particular one.

4. An unknown virus, the complications being due to cocci.

5. Staphylococcal and streptococcal infection—that is, an attenuated pyæmia due to pyogenic cocci (Gustav Singer).

Poynton and Paine have failed to isolate the anærobic bacillus (Achalme's), and have never found any staphylococcus pyogenes aureus ; while Foulerton[10] considers that Achalme's bacillus is the anærobic bacillus enteritidis sporogenes of Klein. He has found vegetations on the mitral valves of rabbits which have not been inoculated with the diplococcus, and such conditions might account for their presence after inoculation, but Poynton and Payne state those obtained in their experiments to have been quite recent.

The Harrogate waters have been rendered more efficient therapeutically by Smith,[11] who shows that nascent sulphur can be driven into the skin by means of the electric current. He tested this property by means of pig's skin and found that the sulphur was passed by the current through the skin and deposited upon a sheet of blotting paper beyond. He claims that this method produces superior results to simple immersion in the sulphur water for eczema, gout, rheumatism, and peripheral neuritis.

The three main theories with regard to scurvy are—

1. Prof. Wright's, viz., that it is due to an acid intoxication of the blood.

2. Lieut. Glen Liston's, viz., that it is dependent upon the presence of the ankylostoma duodenale in the intestine.

3. That due to Jackson and Harley, viz., ptomaine poisoning from eating tainted animal foods.

Lamb[12] criticises these theories in the light of experience gained from 11 cases of scurvy among Hindus in India. He states that there was no marked deficiency in the alkaline foodstuffs of the dietary before the onset of the symptoms. The diet was common to about 1,095 prisoners, and of these only six men developed scorbutic symptoms. There was no diminution in the alkalinity of the blood serum observed when the symptoms were well marked. There was no improvement in the symptoms either as the result of the giving of a diet consisting of abundant alkaline foodstuffs, or on the administration of large doses of lactate of soda. There were present no large numbers of the ova of ankylostoma duodenale in the fæces. Hence his observations tend to show that a condition of acid intoxication is no necessary concomitant, nor is the disease an expression of ankylostomiasis. The amount of meat given to the persons under observation which was always fresh, being killed the same day, was so small as to render the third theory very improbable.

An interesting case is recorded by Box[13] of an attack of scarlet fever in a youth, aged 16, who had hæmophilic tendency and family history. A large extravasation of blood occurred in the floor of the mouth, two attacks of epistaxis and hæmorrhages into the skin, and probably pleura. They all yielded to treatment, and the patient recovered.

After experiments on diet with the subsequent analysis of the excreta for uric acid, Chittenden[14] comes to the conclusion that in man this substance has a twofold origin : one portion coming from the breakdown of the man's own body and so endogenous, and the other portion, usually the larger, is of exogenous origin, coming from the transformation of free and combined purin compounds present in the food.

[8] Brit. Med. Jour., Oct. 12. [9] Brit. Med. Jour., Sept. 21. [10] Ibid. [11] Lancet, Aug. 10. [12] Lancet, Jan. 4. [13] Lancet, Sept. 21. [14] Quar. Med. Jour., Aug.

